# Effect of Erosion on Productivity in Subtropical Red Soil Hilly Region: A Multi-Scale Spatio-Temporal Study by Simulated Rainfall

**DOI:** 10.1371/journal.pone.0077838

**Published:** 2013-10-17

**Authors:** Zhongwu Li, Jinquan Huang, Guangming Zeng, Xiaodong Nie, Wenming Ma, Wei Yu, Wang Guo, Jiachao Zhang

**Affiliations:** 1 College of Environmental Science and Engineering, Hunan University, Changsha, PR China; 2 Key Laboratory of Environmental Biology and Pollution Control (Hunan University), Ministry of Education, Changsha, PR China; 3 College of Resources and Environment, Hunan Agricultural University, Changsha, PR China; Plymouth University, United Kingdom

## Abstract

The effects of water erosion (including long-term historical erosion and single erosion event) on soil properties and productivity in different farming systems were investigated. A typical sloping cropland with homogeneous soil properties was designed in 2009 and then protected from other external disturbances except natural water erosion. In 2012, this cropland was divided in three equally sized blocks. Three treatments were performed on these blocks with different simulated rainfall intensities and farming methods: (1) high rainfall intensity (1.5 - 1.7 mm min^−1^), no-tillage operation; (2) low rainfall intensity (0.5 - 0.7 mm min^−1^), no-tillage operation; and (3) low rainfall intensity, tillage operation. All of the blocks were divided in five equally sized subplots along the slope to characterize the three-year effects of historical erosion quantitatively. Redundancy analysis showed that the effects of long-term historical erosion significantly caused most of the variations in soil productivity in no-tillage and low rainfall erosion intensity systems. The intensities of the simulated rainfall did not exhibit significant effects on soil productivity in no-tillage systems. By contrast, different farming operations induced a statistical difference in soil productivity at the same single erosion intensity. Soil organic carbon (SOC) was the major limiting variable that influenced soil productivity. Most explanations of long-term historical erosion for the variation in soil productivity arose from its sharing with SOC. SOC, total nitrogen, and total phosphorus were found as the regressors of soil productivity because of tillage operation. In general, this study provided strong evidence that single erosion event could also impose significant constraints on soil productivity by integrating with tillage operation, although single erosion is not the dominant effect relative to the long-term historical erosion. Our study demonstrated that an effective management of organic carbon pool should be the preferred option to maintain soil productivity in subtropical red soil hilly region.

## Introduction

Soil erosion, including tillage, wind, and water erosion, is a main type of soil degradation considered as a widespread natural geological phenomenon and a major factor causing the reduction of soil productivity and organic matter content [1–4]. Approximately more than two billion ha of biologically productive land have been irreversibly degraded since 1000 AD [3]. BL Turner concluded that more productive soil may have been irreversibly lost in the past 10,000 years than the amount of soil used for current agricultural production [5]. As global warming adversely affects the environment and extreme rainfall events frequently occur [6], soil erosion continuously poses environmental risks [7]. Therefore, soil erosion and its threat to the environment [4], particularly to food security [8], should be addressed. Adverse effects of erosion on soil productivity have been identified and a qualitative consensus has been obtained [9,10]. However, the factors or mechanisms that determine the soil productivity reduction influenced by soil erosion particularly in a small-scale spatio-temporal study have not been clearly elucidated [11].

Soil water erosion is the displacement of soil from a place where rainfall and runoff originate to another place where water carrying the soil particles flows [3]. Erosion occurs in three distinct stages: detachment; transport/redistribution; and deposition [12]. This process results in the breakdown of structural aggregates [13], excitation of soil organic matter (SOM) decomposition and redistribution of sediments and soil nutrients across the landscape [14–16]. Therefore, erosion depletes soil fertility, reduces the effective rooting depth, and destroys natural resources at different scales [3]. Previous studies showed that the conventional regressors considered responsible for productivity reductions are (1) root growth hindrance by a clayey subsoil or by a pan or bedrock (2), water deficit, and (3) nutrient deficit [11]. For instance, the subtropical red soil hilly region in southern China is an important foodstuff producing area characterized by extensive sloping cropland. This region has suffered from serious water erosion that has severely reduced its soil productivity. Therefore, the changes in soil productivity caused by increasing erosion should be assessed to develop effective measures and implement sustainable agricultural production in this region.

Studies have been performed to detect soil productivity response to erosion, although the effects of erosion on productivity cannot be directly determined by monitoring the evolution of yields on eroding sites through time [11,17,18]. One of the problems in the direct assessment of erosion-productivity relationships is the difficulty in detecting a decline in productivity that results from erosion [17]. This problem is attributed to the effects of soil erosion on productivity of many soils masked by increased inputs particularly by the increased use of nitrogen fertilizer [4]. Hence, researchers used various indirect methods such as simulating erosion by mechanical topsoil removal or desurfacing [17], adding topsoil to eroded soils [20], comparing eroded phases of landscape transects [21], comparing plots with different levels of historical erosion but similar characteristics [22], and establishing simulation models of crop growth response to erosion [11,17]. Most of these approaches have indicated that the response of productivity to soil erosion is a lengthy process [19,23]; therefore, related studies have been based on a large temporal scale [24,25]. Although regressors regulate productivity after suffering from long-term erosion, other important factors show significant dynamics during single rainfall erosion event and immediately affect crop growth of shallow-rooted plants. For instance, soil organic carbon (SOC) and nitrogen, which are mainly enriched on the soil surface and prone to erosion and mineralization [4,26], are redistributed across the sloping cropland during erosion caused by rainfall and runoff [27]. Erosion also breaks down structural aggregates and releases more nutrient resources previously protected in soil. This breakdown may promote nutrient absorption and utilization of crops and thus benefit their growth.

This study aimed to determine the effects of water erosion (including long-term historical erosion and single rainfall erosion event) on soil properties (nutrients) and soil productivity in different farming systems in the red soil hilly region in subtropical China. This study also aimed to describe the regressors or mechanisms that control soil productivity under the stress of water erosion at a small plot scale. The theoretical principles investigated in this study were considered as a regional erosion/productivity model or optimization model. In addition, the results of this study provided guidance to prevent soil productivity reduction that may be of considerable interest given the threats and predictions concerning food security.

## Materials and Methods

### 2.1: Ethics Statement

In this study, soil sampling and sample determinations conducted were permitted by the local authorities (i.e. Soil and Water Conservation Monitoring Station). We also obtained a permission from the local authorities for reporting research results to the public. In addition, the field studies did not involve endangered or protected species.

### 2.2: Study area

**Figure 1:**
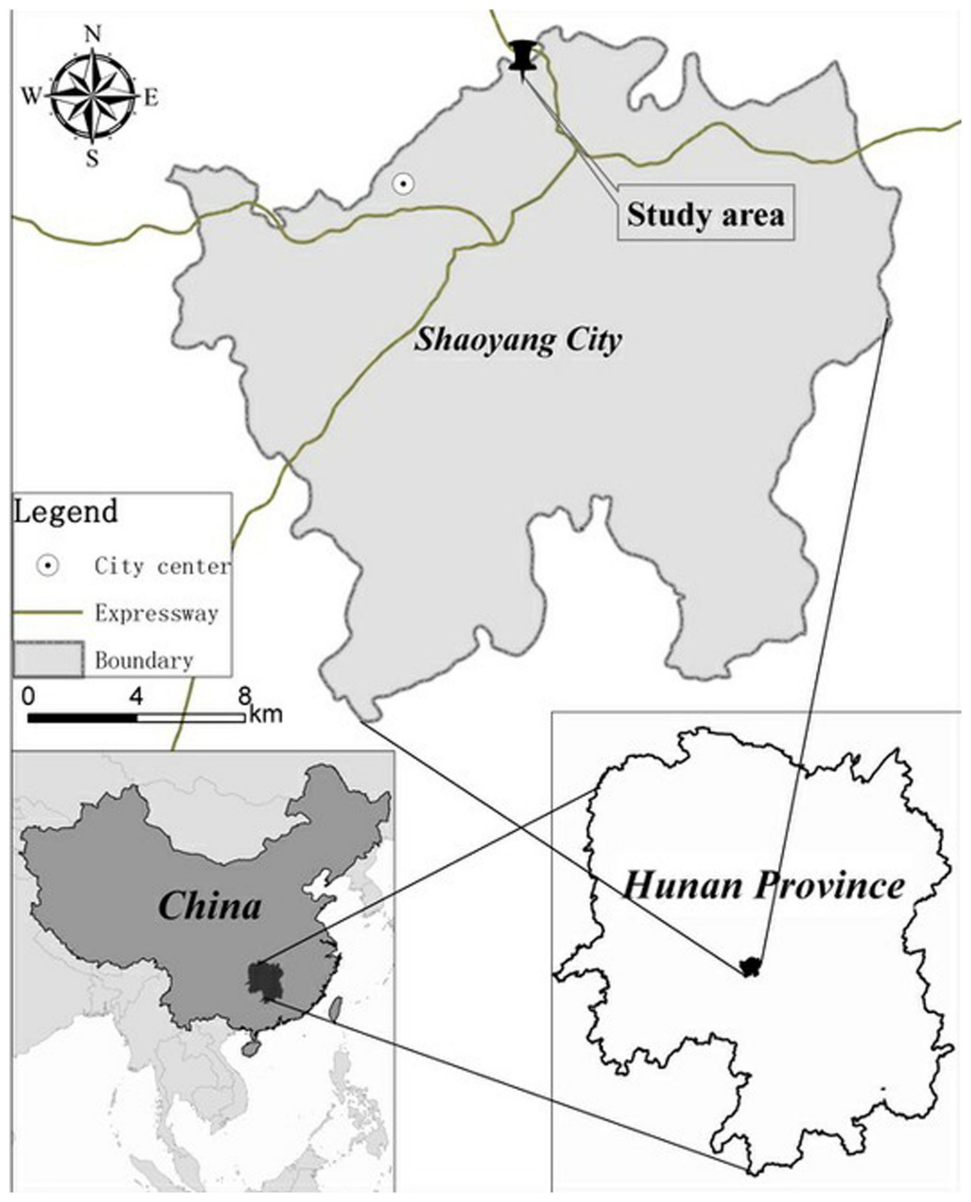
Location of the study area.

This study was performed in Soil and Water Conservation Monitoring Station (N: 25°58′-27°40′, E: 109°49′-112°05′) located in a red soil hilly region at Shuangqing District in Shaoyang City of Hunan Province, China (Fig. **1**). The altitude in the station ranges from 231.18 m to 276.63 m above sea level. The station consists of three typical catchments with a total size of 47.2 km^2^. It has a sub-tropical monsoon climate, an average annual rainfall of 1327.5 mm, and an average annual temperature of 17.1 °C. The main soil type is quaternary red clay, which was classified as Ultisols by the U.S. Soil Taxonomy. The area is characterized by diversified land use patterns, including sloping cropland, woodland, garden, and terrace. Sloping cropland, as the dominant land use pattern, is mainly used to plant *Polygonatum odoratum* (Mill.) Druce under chisel-plow tillage. In summer with high temperature and frequent high-intensity thunderstorms, this area is subjected to serious water erosion.

### 2.3: Study design and sample collection

**Figure 2:**
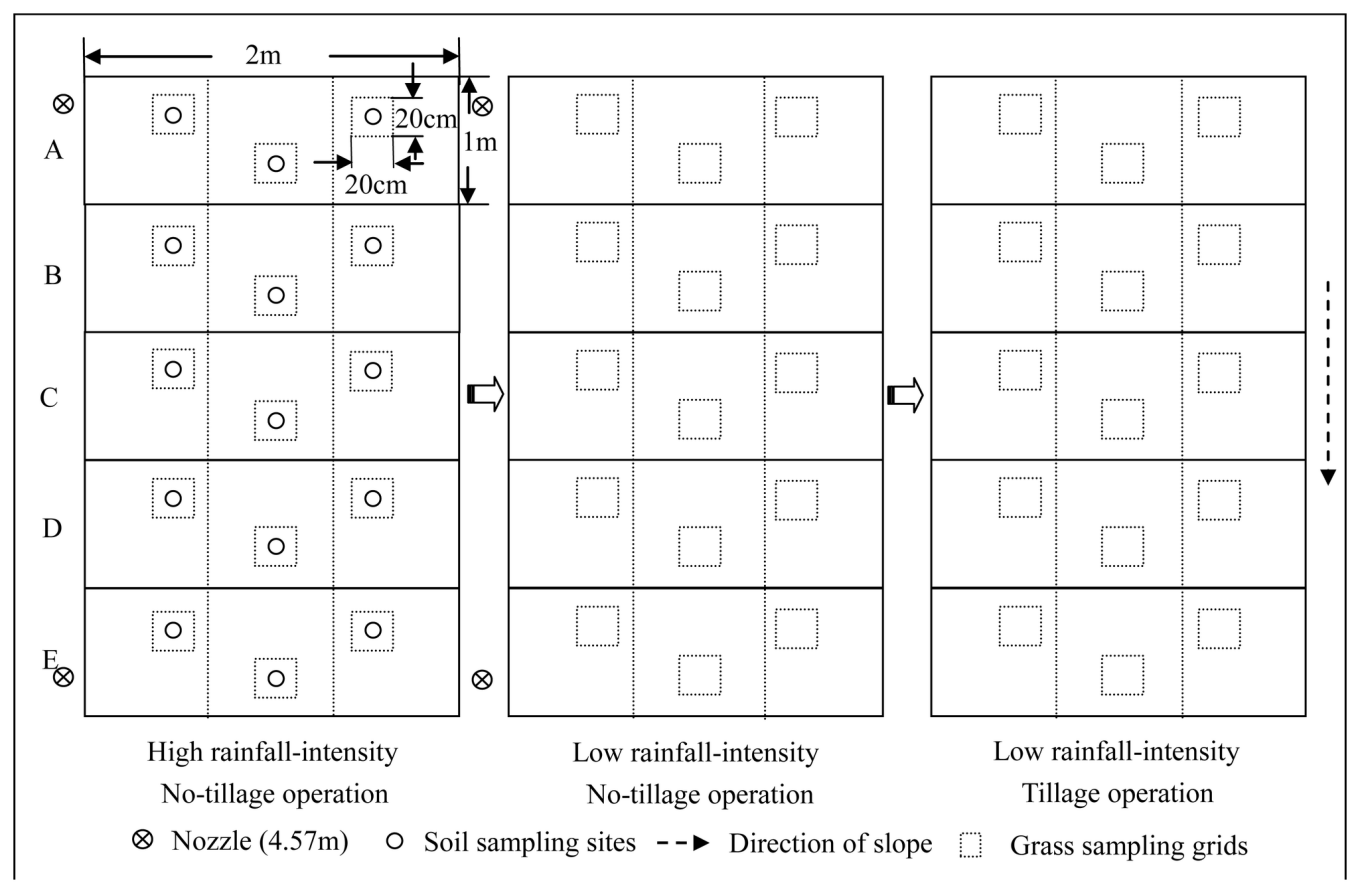
Design of blocks and sampling strategy of the simulated rainfall and grass planting experiments.

A typical cropland, with a slope gradient of approximately 10%, was collected in October 2009 from a long-term planting system (established in the 1980s) in the station. This cropland was remediated to ensure uniformity of the soil properties in the initial state. Afterward, this cropland was protected from other external disturbances except natural water erosion. The simulated rainfall and grass planting experiments were performed in October 2012. Before the simulated rainfall began, three blocks (2 m × 5 m) were obtained from the cropland and used as the rainfall plots with frames made of aluminum metal sheet; the residue was then removed from the surface of these soil plots. Three treatments were performed in the three plots with different simulated rainfall intensities and farming methods: (1) high rainfall erosion intensity, no-tillage operation; (2) low rainfall erosion intensity, no-tillage operation; and (3) low rainfall erosion intensity, tillage operation (Figure **2**). For the simulated rainfall experiments, rainstorms were applied using a rainfall simulator with a SPRACO cone jet nozzle mounted on the top of fixed stand pipes (height of 4.57 m) located at the borders of the plots (Figure **2**). The median drop size was 2.4 mm with a uniformity of 89.7%. To generate varying degrees of water erosion, we designed rainfall intensities of 0.5 mm min^−1^ to 0.7 mm min^−1^ and 1.5 mm min^−1^ to 1.7 mm min^−1^, which represent low and high intensities in this study, respectively. Each simulated rainfall lasted for 1 h and the rainfall plots were sampled in the surface soil (0 mm to 10 mm) for laboratory analysis. After soil sampling, all of the rainfall plots were carefully loosen using a hoe; perennial ryegrass (*Lolium perenne*) seeds were evenly sown on the plots. Plot tillage or loosening was conducted according to the following criteria: (i) minimal soil disturbance and (ii) avoiding soil lateral exchange along the direction of the slope. The first irrigation was applied approximately 5 d after sowing, and other irrigations were provided at an interval of approximately 2 d. After a 30-day culture, the perennial ryegrass was harvested to estimate the soil productivity.

Soil sampling was conducted before and immediately after the simulated rainfall. Each rainfall plot was divided in five equivalent subplots, namely, A, B, C, D, and E (2 m width  1 m length) along the slope and separately sampled. The five equally sized subplots along the slope of each plot could quantitatively characterize different levels of the effects of historical erosion during the last three years. After long-term erosion, these five subplots suffered from different types of historical erosion or deposition, which profoundly affected the soil productivity of the whole sloping cropland. Three separately arranged grids (20 cm  20 cm) were chosen as three replicates in each subplot (Figure **2**). Soil sample was obtained using a corer (70 mm diameter) from the top 10 cm in each grid. The missing parts of the sampling sites were filled and carefully leveled to reduce the effects of soil sampling on the following planting experiments. All of the soil samples were labeled and sealed in air-tight Ziplock bags and immediately stored at -20 °C before use. Grass biomass was separately sampled in each subplot similar to the approach used for soil sampling. In each grid, all of the grass strains were gently uprooted, washed with water, dried at 60 °C for 48 h, and weighed to determine the aboveground biomass (AB) and root biomass (RB). AB was determined by clipping the plants at a ground level. RB was determined as the discrepancy of total biomass and AB.

### 2.4: Soil sample analyses

Soil particle size distribution analysis was applied using the pipette method to determine the clay fraction (CF) [28]. Soil bulk density (BD) was determined from oven-dried undisturbed cores as the mass per volume of dried soil. SOC was determined using the K_2_Cr_2_O_7_ titration method after digestion [29]. Soil TN and soil total phosphorus (TP) were determined by Kjeldahl and Kara methods [30,31], respectively. Three replicates were used in the analysis.

### 2.5: Statistical analysis

We established five equivalent subplots in each rainfall plot to represent varying degrees of historical erosion and to detect the effects of historical erosion characteristic on soil productivity. Therefore, historical erosion was introduced to the analysis model as a nominal variable. The whole data set was categorized in three sets based on the variables: soil productivity data (AB and RB: two quantitative variables), environmental data including soil properties (BD, SOC, TN, TP, C/N, N/P, and CF: seven quantitative variables), historical erosion (a nominal variable), farming methods (a nominal variable), and rainfall intensity (a nominal variable). Direct multivariate analyses (e.g., redundancy analysis (RDA)) were widely used to relate changes in dependent variable (s) to changes in the environment, and provide statistical tests for those correlations [32,33]. Using Canoco (version 4.5), we performed RDA to quantify the effects of the environmental data on soil productivity dynamics in no-tillage and low rainfall erosion intensity systems. Partial RDA was also performed to extract the variation in the soil productivity influenced by each of the explanatory variables (i.e., environmental data) and shared by these data (set) [34]. The soil productivity variables were standardized and centered before the partial RDA analysis. The forward selection procedure of the program was performed to determine the variables with a significant influence on soil productivity. The selection procedures ended when the variables were not significant. Monte Carlo reduced model tests with 499 unrestricted permutations were used to evaluate the significance of the first canonical axis and all of the canonical axes.

## Results

### 3.1: Variation in soil properties

Given that the initial soil properties of the selected sloping land were homogeneous and all of the three blocks obtained from this land suffer from similar external stress during the three-year abandoned period, the soil properties of the three blocks should remain similar before the simulated rainfall. Therefore, we randomly measured two factors, namely, SOC and BD of the soils sampled before simulated rainfall to verify the aforementioned inference. No significant difference (*P* > 0.05) was found among the three blocks in any of the two measures (data not shown). Table **1** lists the spatial trends of the measured soil properties of each plot and the discrepancy between different treatments after the simulated rainfall. Significant differences (*P* < 0.05) were found in some soil properties between different treatments particularly between no-tillage and tillage plots. All of the measured nutrient elements (i.e., SOC, TN, and TP) showed significant differences (*P* < 0.05) between no-tillage and tillage plots after low-intensity rainfall. No significant differences (*P* > 0.05) were observed between high and low rainfall intensity operations in no-tillage systems. In addition, all of the measured soil properties exhibited a high spatial variation in each simulated rainfall plot. The coefficient of variation was generally ranked as follows: TP > TN > SOC > BD > CF.

**Table 1:** Spatial trends of soil properties within each block and their discrepancy between different treatments after a simulated rainfall.

	SOC (g C kg ^-1^ dry soil)		TN (g N kg ^-1^ dry soil)		TP (g N kg ^-1^ dry soil)		BD (g/cm^3^ dry soil)		CF (%)	
Treatments	Mean	CV	Mean	CV	Mean	CV	Mean	CV	Mean	CV
High rainfall intensity, no-tillage	7.627^a^	34.35	0.780^a^	38.76	0.673^a^	49.85	1.590^b^	10.81	33.683^a^	3.62
Low rainfall intensity, no-tillage	7.809^a^	34.07	0.820^a^	36.96	0.710^a^	48.43	1.753^a^	10.26	33.163^a^	6.44
Low rainfall intensity, tillage	6.927^b^	36.95	0.520^b^	31.14	0.412^b^	31.72	1.772^a^	4.10	33.955^a^	4.30

Values with the same letters are not significantly different at P < 0.05 level as determined by an LSD test in MANOVA model by SPSS 18 version, *n* = 15. CV = coefficient of variation; SOC = soil organic carbon; TN = total nitrogen; TP = total phosphorus; BD = bulk density; CF = clay fraction.

### 3.2: Effects of erosion intensity on soil productivity in no-tillage systems

**Figure 3:**
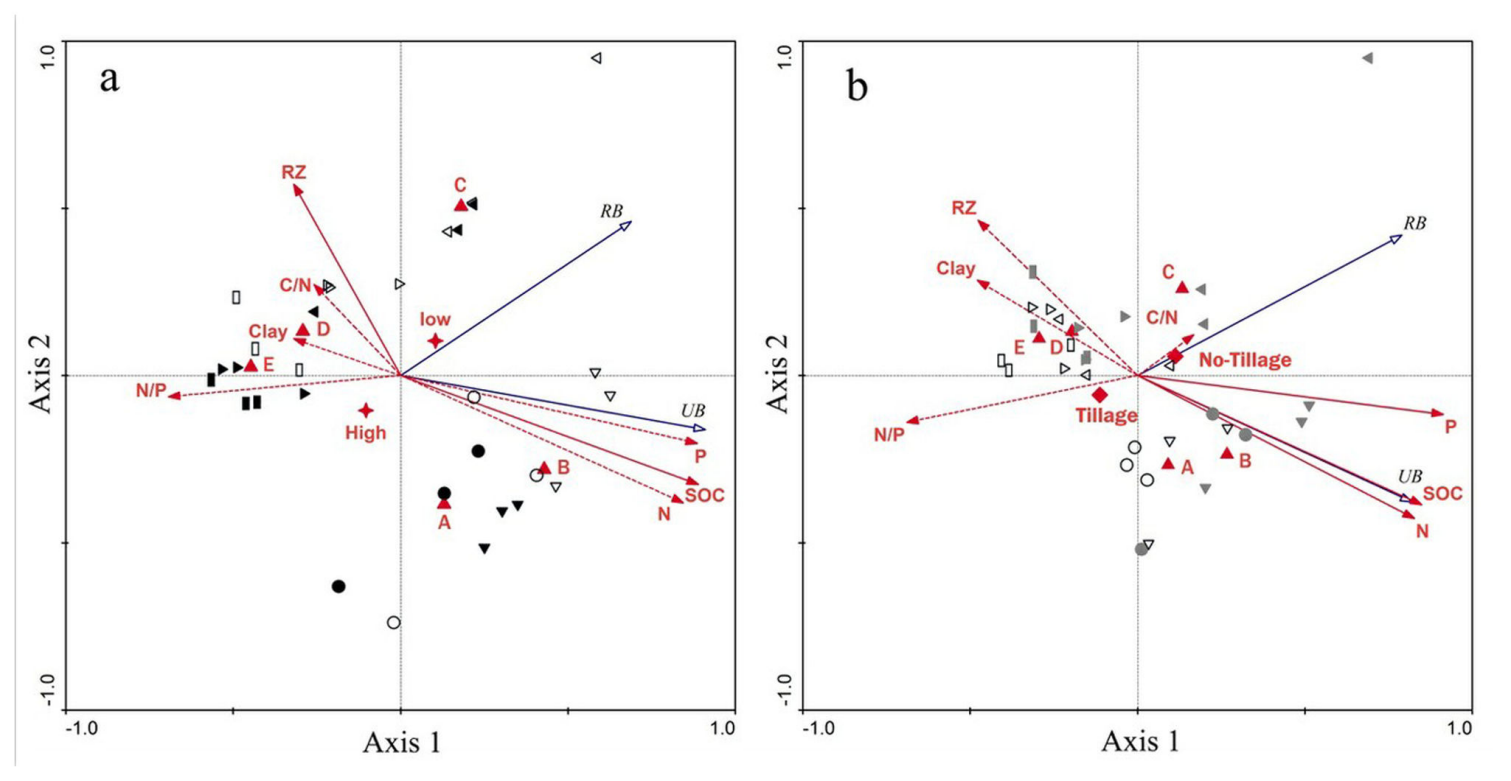
Redundancy analysis of soil productivity data in (a) different erosion intensities with no-tillage operation and (b) same erosion intensity but different farming method systems. Significant soil variables and supplementary parameters are indicated by solid lines with filled arrows and dotted lines with filled arrows, respectively. Soil productivity variables are indicated by solid lines with unfilled arrows. Samples from subplots A, B, C, D, and E are represented by circle, inverted triangle, left triangle, right triangle, and box, respectively. Black-filled symbols in Figure 3a refer to the samples from high rainfall intensity treatment, and unfilled symbols refer to the samples from low rainfall intensity treatment. Gray-filled symbols in Figure 3b refer to the samples from no-tillage treatment, and unfilled symbols refer to the samples from tillage treatment.

Under different erosion-intensity and no-tillage conditions (DENT), the correlation structure between soil productivity and environmental data is summarized in Figure **3a**. The eigenvalues of the first axis and the second axis were 0.717 and 0.085, respectively. The variables that were highly correlated with axis 1 included SOC (*r* = 0.8175, *P* < 0.001), TP (*r* = 0.8146, *P* < 0.001), TN (*r* = 0.7763, *P* < 0.001), and N:P ratio (*r* = −0.6373, *P* < 0.001). All of the environmental variables could accounted for 80.2% of the variation in soil productivity (Monte Carlo permutation test with 499 permutations, *P* = 0.002). This study aimed to identify the factors causing the changes in soil productivity. The parameters that best described the most influential gradients were identified by forward selection. Forward selection indicated that SOC (*P* = 0.016) and BD (*P* = 0.01) exhibited the highest significant amount of variation in the seven measured soil properties. SOC and BD could account for 66.3% of the soil productivity variation (Monte Carlo permutation test with 499 permutations, *P* = 0.002). In addition, RDA on soil productivity data limited by historical erosion data accounted for 61.4% (*P* = 0.002) of the productivity variation. However, no significant explanation was detected when the RDA model was solely limited by erosion-intensity data (*P* = 0.076). All of the significant explanatory variables could explain 74.9% of the soil productivity variation (Monte Carlo permutation test with 499 permutations, *P* = 0.002).

**Figure 4:**
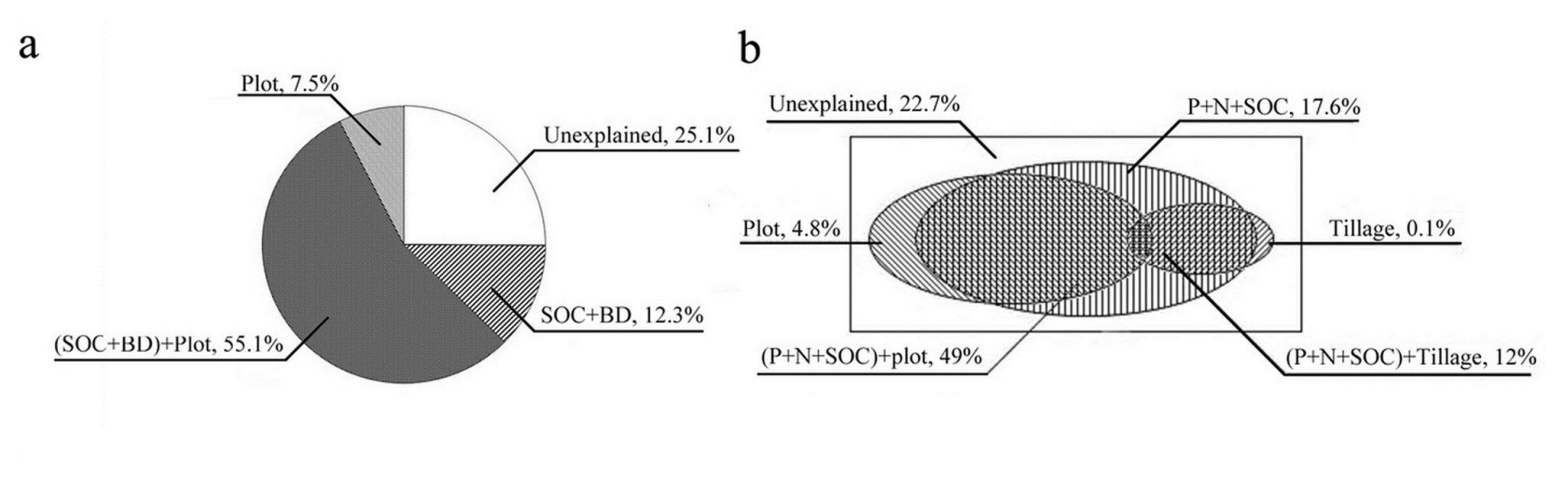
Variance partitioning with partial RDA of soil productivity based on significant soil variables, historical erosion (plot), and farming method data (set) in (a) different erosion intensities with no-tillage operation and (b) same erosion intensity but different farming method systems. Monte Carlo permutation test was performed on each set without the effect of the other by freely permuting samples (499 permutations).

Partial RDA was used to extract the variation in soil productivity of each of the significant explanatory variables or their data sets without affecting another variable as well as the variation caused by such variables. The variation in soil productivity explained by the data set of the significant soil variables (i.e., SOC and BD) without the second variable was also significant (*P* = 0.008). By contrast, the historical erosion data revealed different results (*P* = 0.106). For each explanatory variable or data set, the variation presented in Figure **4a** was caused by the variables without the shared variation. The data set of the significant soil variables and historical erosion data accounted for 12.3% and 7.5% of the variation in soil productivity, respectively. The variation shared by these two data sets was 55.1%. A total of 25.1% of variation in soil productivity could not be explained by the measured and introduced variables. To investigate the mechanism by which historical erosion and single erosion event under different farming methods affect soil productivity, we obtained the interactive explanation of these nominal variables and each significant soil properties (Table **2**). The variation shared by historical erosion data and SOC was 49.1%, whereas only 0.07% of the variation could be shared by historical erosion and BD.

**Table 2:** Shared variation of each significant soil variable and historical erosion (plot), erosion intensity, and farming method data.

	Different erosion intensities with no-tillage operation		Different farming methods with same erosion intensity	
Soil variables	Plot	Erosion intensity	Plot	Farming methods
SOC	49.1%	NS	41.3%	5.6%
TN	NS	NS	42.2%	6.8%
TP	NS	NS	36.9%	8.6%
BD	7.0%	NS	NS	NS

NS = no significant shared variation; SOC = soil organic carbon; TN = total nitrogen; TP = total phosphorus; BD = bulk density.

### 3.3: Effects of erosion on soil productivity under different farming methods

Under the same erosion intensity but different farming methods (SEDF), the correlation structure between soil productivity and environmental data is summarized in Figure **3b**. The eigenvalues for the ﬁrst and second axes were 0.648 and 0.159, respectively. Soil variables that were highly correlated with axis 1 included TP (*r* = 0.828, *P* < 0.001), SOC (*r* = 0.769, *P* < 0.001), TN (*r* = 0.749, *P* < 0.001), N:P ratio (*r* = - 0.625, *P* < 0.001), CF (*r* = - 0.434, *P* < 0.02), and BD (*r* = −0.432, *P* < 0.02). All the environmental variables in combination could explain 80.7% of the variation in soil productivity (Monte Carlo permutation test with 499 permutations, *P* = 0.002). The ‘forward selection’ procedure indicated that the soil variables explaining the largest statistically significant amount of variation were TP (*P* = 0.002), SOC (*P* = 0.006), and TN (*P* = 0.01). All of the three significant soil variables could together explain 72.4% of the variation in productivity (Monte Carlo permutation test with 499 permutation, *P* = 0.002). In addition, RDA on soil productivity data solely limited by tillage practices and historical erosion data showed that the two RDA models statistically accounted for 12.1% (*P* = 0.032) and 53.8% (*P* = 0.002) of the productivity variation, respectively. All of the significant explanatory variables could account for 77.2% of the soil productivity variation (Monte Carlo permutation test with 499 permutations, *P* = 0.002).

Partial RDA showed that the variation in soil productivity explained by the significant soil variables without the second axis reached a maximum of 17.6% (*P* = 0.002). By contrast, no significant individual explanations for this measure were observed in historical erosion (*P* = 0.403) and tillage practices data (*P* = 0.902). The variation shared by soil property data and historical erosion as well as tillage practices data were 49.0% and 12.0%, respectively. Approximately 22.7% of variation in soil productivity could not be explained by these significant variables (Figure **4b**). Table **2** shows the interaction between each significant soil variables and historical erosion as well as soil tillage practice data. The shared explanations influenced by the significant soil variables and historical erosion data were generally higher than those by the significant soil variables and tillage practice data. The shared explanations developed by each significant soil variables and historical erosion data were generally ranked as follows: TN (42.2%) > SOC (41.3%) > TP (36.9%). The shared explanations developed by each significant soil variable and tillage practice data were generally ranked as follows: TP (8.6%) > TN (6.8%) > SOC (5.6%).

## Discussion

Soil erosion is an important manifestation of soil degradation because it involves the physical removal of soil and the contained plant nutrition vertically and/or horizontally, thereby degrading soil quality and reducing productivity [1]. Studies involving erosion-productivity assessment insisted that the effects of erosion on productivity should be attributed to long-term accumulation and single erosion event could not impose any significant constraints on soil productivity [23,35]. In this study, two sets of experiments (i.e., DENT and SEDF) were performed to investigate the mechanism and the extent by which single rainfall erosion event affects soil productivity. Under DENT, RDA analysis showed that erosion intensity was not statistically responsible for the variation in soil productivity in these sloping lands. This result suggested that no significant effects of single rainfall erosion event on soil productivity were observed under no-tillage practice regardless of erosion intensity. This phenomenon profoundly indicated that this practice could benefit soil productivity under the stress of single rainfall erosion. No-tillage is a combination of ancient and modern agricultural practices [36]. The rapidly increasing use of this practice is partially due to its active function in maintaining soil productivity under the exacerbated stress induced by erosion. A large amount of plant nutrition (e.g., SOM) is physically protected within soil aggregates in undisturbed soil. The destruction of this soil caused by erosion/slaking could exacerbate the loss of such nutrients in the soil aggregates. Under no-till conditions, the dense soil structure was more stable. In combination with high viscosity and corrosion resistance of the red soil [37], soil aggregates were no longer slaked and a dense soil surface crust formation ceased [38]. Many studies have found that no-tillage can improve soil resistance to water erosion by creating indurated crusts and preventing rill generation [23,39]. Less soil disturbance could improve the ability of the soil to retain nutrients and reduce the adverse effects of erosion on productivity. However, the opposite was true under SEDF; the till practice data (a nominal variable) significantly accounted for 12.1% of the soil productivity in the single erosion event. This finding indicated that plowing before seeding could remarkably stimulate the action of a single erosion event on soil productivity. In addition, no-till treatment plots indicated higher soil productivity than the plowing plots. This result is consistent with our pervious analysis because no-till soils are resistant to erosion. Artificial destruction by plowing could seriously destroy the soil structures (soil aggregates), thereby exposing the plant nutrition previously protected within the soil aggregates to erosion attack. Therefore, we argued that the common view that the short-term effects of erosion on soil productivity by single rainfall event could be ignored should be challenged. Therefore, we should give considerable attention to the interaction of tillage practices and single erosion events with soil productivity when investigating soil erosion-productivity relationship. The majority of the agricultural methods in the subtropical red soil hilly region in south China still use plowing and loosening before seeding. Thus, effective conservation measures should be investigated to reduce or eliminate the negative effects of single rainfall event particularly when extreme precipitation events are continuously induced by climate change in this region.

Since the establishment of the experimental cropland in 2009, it has been protected from other external disturbances except natural water erosion. Water erosion leads to great spatial heterogeneity of most soil properties of the sloping land particularly the surface soil. Quinton et al. have found that the selective removal of erosion could redistribute plant nutrients and soil particles within the landscape, thereby resulting in diverse soil properties for crop growth [10]. As a shallow-rooted crop that relies strongly on the conditions of surface-soil, *L. perenne* is considered sufficiently sensitive to the topsoil disturbance induced by erosion. We found that the cumulative effects of historical erosion controlled the soil productivity under DENT and SEDF. The plots (nominal variable) significantly showed 61.4% and 53.8% of variations in soil productivity under DENT and SEDF, respectively. This finding is consistent with that in other studies [11,40], in which the cumulative effect of long-term erosion rather than single erosion event is the dominant stress responsible for soil productivity reduction.

The mechanism by which erosion (including historical erosion and a single erosion event) affects soil productivity by various soil properties under different farming conditions should be understood for the development of an effective soil management strategy. Previous study on the relationship of crop productivity to erosion found that the soil variables affecting the shape of the erosion-productivity response curve were water deficit, physical root hindrance, and nutrient deficit [11]. As explained earlier, the pre-selected shallow-rooted plants and the loosening operation before seeding may largely eliminate the reduction of productivity because of physical root hindrance. Given that regular irrigation was applied approximately 5 d after sowing and then at an interval of 2 d, water deficit could also be excluded in this study. The heterogeneity of the soil properties affected by erosion particularly nutrient redistribution and loss induced by single erosion event may directly contribute to the variation in soil productivity on the slope. Indeed, the results showed that most of the variations affected by the plots or the till practice data set were the combined actions of these data (set) and the soil properties data set. In fact, the nutrients that originate from organic matter are often situated at or near the surface and decreases non-linearly with soil depth [11]. The highest reductions in nutrient availability occur when the first few centimeters of topsoil are removed; therefore, productivity reductions are possibly the highest when initial soil is removed. The importance of SOC in regulating soil productivity is well known [41–43]. Our data indicated that SOC was a major limiting variable that influenced the soil productivity under DENT and SEDF. This result suggested that the effective management of the organic carbon pool should be the preferred option in the subtropical red soil hilly region. Given the high leaching and decomposition rate, SOC content is rather low in the subtropical red soil hilly region in south China [37]. This low content may cause a nutrient deficit for crop growth; therefore, organic matter carbon is considered as a major productivity-dependent factor. Interestingly, although all of the plots were loosened after the simulated erosion, our data indicated that BD was a strong predictor of soil productivity in DENT. Many studies have found that soil penetration and erosion resistance are consistent with BD [44,45]. Considering this finding, we could conclude that BD may indirectly influence soil productivity by preventing soil erosion and nutrient loss during a long-term natural process. Tillage systems are considered susceptible to water erosion, and soil nutrient availability for crop growth may be strongly affected by this stress. Therefore, we expected the dynamics of soil C, N, and P induced by single erosion event might function in determining soil productivity on the sloping land. We found that SOC, TN, and TP were strong predictors of soil productivity under SEDF. This result indicated that the significant influence of single erosion event on soil productivity could be partially explained by the excited migration or loss of these nutrients as a result of applying tillage operations [46]. We also detected that the shared level between tillage practice data and each of the three significant factors was almost similar. This finding is consistent with Lal and Yang who suggested that the dynamic and function of these nutrient elements are generally coupled to one another in geochemical and biological processes [37,41]. In addition, the variations shared by plot data and each of the three significant factors were evidently higher than the variations shared by tillage practice data and each of these factors, respectively. Therefore, we argued that the effects of long-term historical erosion on soil properties should have a major function in erosion-productivity relationships relative to a single erosion event regardless of tillage system application.

## Conclusion

The effects of water erosion including long-term historical erosion and a single rainfall–erosion event on soil properties and productivity in different farming systems in the subtropical red soil hilly region in south China were investigated. Our data showed that the effects of long-term historical erosion significantly explained most of the variation in soil productivity in no-tillage and tillage systems. This finding indicated that the effects of long-term water erosion should have a major function in inducing soil heterogeneity and variations in soil productivity relative to single rainfall erosion event. This finding is consistent with the general conclusion of many previous studies. However, our data indicated that the intensities of single simulated erosion exhibited no significant effects on soil productivity in no-tillage systems. By contrast, different farming operations indeed induced a statistical difference in soil productivity under the same single-erosion intensity. On one hand, this finding suggested that no-tillage practice could improve soil resistance to water erosion and sustain soil productivity. On the other hand, this finding provided strong evidence that single erosion event could also cause significant limitations on soil productivity by integrating with tillage operation, which is largely ignored in previous studies from an agricultural perspective. This study demonstrated that SOC, which was detected at a low content in the red soil, was the major limiting variable that influenced soil productivity. This result suggested that an effective management of the organic carbon pool should be the preferred option in subtropical red soil hilly region. Notably，we detected that most of the explanations of long-term historical erosion related to the variation in soil productivity arose from the sharing with SOC. This result suggested that the redistribution or loss of SOC induced by long-term natural erosion was the dominant mechanism that regulates the erosion-productivity relationship. In tillage system, SOC, TN, and TP were found to be the regressors of soil productivity. Therefore, we concluded that single erosion event could change the pattern of soil productivity by changing the dynamics of these three nutrient elements of the sloping land. However, considering the uncertainties of natural rainfall (e.g., intensity, duration and spatial uniformity), simulation rainfall experiment may be an ideal scenario. Therefore, further prospective studies are suggested to investigate erosion – productivity relationship by natural observations.
